# An Explainable Deep Learning Framework for Musculoskeletal Abnormality Detection

**DOI:** 10.3390/bioengineering13101177

**Published:** 2026-10-09

**Authors:** Saeed Ur Rehman, Muhammad Saad, Anwar Ali, Muhammad Awais

**Affiliations:** 1 School of Computer Science, University of Hull, Hull HU6 7RX, UK; 2 Faculty of Science and Engineering, Swansea University, Swansea SA1 8EN, UK; 3 Department of Computer Science, College of Computer, Qassim University, Buraydah 52571, Saudi Arabia

**Keywords:** musculoskeletal radiographs, musculoskeletal abnormality detection, deep learning, transfer learning, explainable artificial intelligence, Grad-CAM, weak localization, computer-aided diagnosis, MURA

## Abstract

Accurate detection of musculoskeletal abnormalities from radiographs remains challenging because abnormal findings can be subtle and radiographic appearance may vary considerably across examinations. This study aimed to develop and systematically evaluate a deep learning framework incorporating Grad-CAM-based visual attribution for automated musculoskeletal abnormality detection using the publicly available Musculoskeletal Radiographs (MURA) dataset. Four transfer learning architectures, namely DenseNet201, EfficientNetV2-S, ConvNeXt Tiny, and Swin Transformer Tiny, were evaluated for musculoskeletal abnormality classification. The framework incorporated Grad-CAM-based attribution and activation-based weak localization to highlight image regions associated with model predictions without requiring bounding-box annotations during training and a sensitivity-oriented decision threshold to prioritize the detection of abnormal examinations. Model performance was assessed using recall, precision, F1-score, area under the receiver operating characteristic curve (AUC), and confusion matrix analysis. A targeted decision-threshold ablation analysis compared the sensitivity-focused threshold of 0.40 with the conventional 0.50 baseline using the same validation-set probabilities. Previously published results were reviewed to provide contextual information rather than establish comparative superiority. EfficientNetV2-S achieved the highest precision (0.925), F1-score (0.892), and AUC (0.956) on the internal validation set among the evaluated architectures. DenseNet201 achieved the highest recall of 0.876. Previously published performance metrics were reviewed to contextualize the results of the present study. However, differences in datasets, anatomical coverage, preprocessing procedures, and evaluation protocols preclude direct conclusions regarding comparative superiority. At the sensitivity-focused threshold of 0.40, EfficientNetV2-S obtained an accuracy of 0.896 and an F1-score of 0.892 on the internal validation set. Lowering the threshold to 0.40 improved sensitivity from 0.754 to 0.861 and decreased false-negative predictions from 1092 to 618 when compared to the traditional threshold of 0.50. Alongside this improvement, false-positive predictions increased from 150 to 307 and specificity decreased from 0.966 to 0.931. Grad-CAM visualizations and activation-derived bounding boxes provided qualitative information about spatial attribution. However, their correspondence with pathological findings was not independently validated using expert annotations or quantitative localization metrics. On the independent held-out test set, EfficientNetV2-S achieved a recall of 0.826, precision of 0.886, F1-score of 0.855, and AUC of 0.927. The independent evaluation demonstrated lower performance than the internal validation results, highlighting the importance of separating model optimization from final performance assessment. The findings demonstrate the feasibility of integrating transfer learning, Grad-CAM-based visual attribution, activation-based weak localization, and sensitivity-oriented decision-making within a unified framework for musculoskeletal abnormality classification. Although the framework demonstrated promising classification performance, the visual attribution results remain exploratory and do not establish pathological localization accuracy or clinical interpretability. Further evaluation using external clinical datasets, expert-annotated pathological regions, and radiologist assessment is required to establish its generalizability, explanation validity, and potential clinical utility.

## 1. Introduction

Medical imaging plays a fundamental role in modern healthcare, with musculoskeletal radiography serving as the primary imaging modality for diagnosing bone fractures and other skeletal abnormalities. Accurate detection of musculoskeletal abnormalities from radiographs is essential for effective clinical decision-making, as delayed or missed diagnoses may result in inappropriate treatment, prolonged disability, and increased healthcare burden [1]. Nevertheless, interpreting musculoskeletal radiographs remains challenging because abnormalities may be subtle and image appearance can vary considerably across anatomical regions and patients [1]. Consequently, artificial intelligence (AI)-based computer-aided diagnostic systems have attracted increasing attention as decision-support assisting radiologists in image interpretation and potentially improving diagnostic accuracy [2].

Recent advances in deep learning have significantly improved the performance of automated medical image analysis by enabling models to learn complex hierarchical image features directly from data, eliminating the need for handcrafted feature extraction [2]. Among deep learning approaches, convolutional neural networks (CNNs) have become the dominant architecture for medical image classification because of their ability to capture local spatial features and achieve high diagnostic performance across a wide range of imaging applications [2,3]. More recently, Vision Transformer-based architectures, such as Swin Transformer, have demonstrated strong contextual modeling by capturing long-range dependencies through self-attention mechanisms. However, their performance often depends on the availability of large-scale training datasets [4]. Since labeled medical imaging datasets are often considerably smaller than large-scale natural image datasets such as ImageNet, transfer learning has become a widely adopted strategy for improving model performance in medical image analysis [2,5].

Despite the remarkable diagnostic performance achieved by deep learning models, their adoption in routine clinical practice remains limited because many models operate as “black-box” systems, providing predictions without clearly explaining the underlying reasoning. Consequently, Explainable Artificial Intelligence (XAI) has emerged as an important research direction for improving model transparency and enabling clinicians to better understand and verify AI-assisted diagnostic decisions [6,7]. Among the various XAI techniques, Gradient-weighted Class Activation Mapping (Grad-CAM) has become one of the most widely adopted visualization methods, as it highlights the image regions that contribute most to the model’s prediction, thereby providing intuitive visual explanations of the decision making process [6]. Beyond model interpretation, weakly supervised localization techniques further enhance the practical value of deep learning systems by enabling approximate localization of abnormalities without requiring expensive pixel-level or bounding-box annotations during training [8,9]. Together, explainability and weak localization improve the transparency and clinical trustworthiness of computer-aided diagnostic systems, making them more suitable for supporting radiologists in real-world medical imaging applications [6,7]. Integration of Grad-CAM-based explainability with an activation-derived region visualization procedure helped to highlight image regions contributing to model predictions. This approach generates approximate bounding boxes around highly activated regions without requiring lesion-level annotations during model training. These visualizations provide exploratory spatial attribution rather than quantitatively validated pathological localization. Consequently, the generated bounding boxes should be interpreted as activation-derived regions of interest rather than confirmed lesion locations. Another important consideration in medical abnormality detection is the classification threshold. A conventional probability threshold of 0.50 may not be optimal when minimizing false-negative predictions is a priority. A sensitivity-focused threshold can increase the likelihood of identifying borderline abnormal cases, although potentially at the cost of additional false positives [10,11]. The present study therefore incorporates a sensitivity-oriented thresholding strategy together with classification and visual interpretation.

## 2. Literature Review

Data-driven artificial intelligence approaches have increasingly been applied to healthcare-related problems, including analysis of large-scale healthcare data [12,13]. Table 1 demonstrates the progressive development of deep learning approaches for musculoskeletal abnormality detection. Rajpurkar et al. [1] established the MURA benchmark using DenseNet-169 and demonstrated the feasibility of automated abnormality classification, achieving a recall of 0.815 and an AUC of 0.929. This work provided an important foundation for subsequent research on musculoskeletal abnormality classification and incorporated class activation maps for qualitative visual interpretation. He et al. [14] proposed a calibrated deep-learning ensemble for abnormality detection in musculoskeletal radiographs using the MURA dataset, demonstrating the potential of ensemble architectures and class activation maps for both classification and visual interpretation. Building on this direction, the two-stage VGG16 approach [15] investigated a more structured classification strategy using MURA. However, the reported F1-score of 0.688 indicates comparatively limited classification performance. More importantly, the approach remained focused on abnormality classification rather than providing visual explanations or localization of suspected abnormalities.

The performance values presented in Table 1 are reproduced from the respective publications and are intended solely to provide context regarding previously reported results. The studies differ in their anatomical coverage, dataset composition, preprocessing procedures, and evaluation protocols. Consequently, these numerical results do not constitute a controlled comparison and should not be interpreted as evidence of the superiority of any particular approach. A direct comparison would require evaluation of the respective methods using a common, predefined test set and consistent experimental procedures. The study in [16] introduced a more complex hybrid architecture combining ResNet50, DenseNet121, VGG16, an autoencoder, and squeeze-and-excitation blocks. The method achieved a recall of 0.860 and an AUC of 0.940, demonstrating the potential of feature fusion and hybrid architectures. However, the study used both MURA and LERA datasets, which makes direct comparison with approaches trained exclusively on MURA more difficult.

The two-stage DenseNet201 framework [17] achieved a precision of 0.870, an F1-score of 0.790, and an AUC of 0.790, with a recall of 0.690. Although the study demonstrated the applicability of DenseNet201 to musculoskeletal radiographs, its reported evaluation was focused on shoulder classification. Therefore, its results may not directly represent performance across the complete range of anatomical regions contained in MURA.

The approach presented in [18] reported substantially higher performance, with recall, precision, and F1-score of 0.996, 0.989, and 0.992, respectively. However, these results were obtained using only the shoulder subset of MURA together with same-domain transfer learning and feature fusion. Consequently, the reported performance should not be directly compared with studies evaluating all MURA anatomical regions.

Compared with these studies, this work achieves a recall of 0.861, precision of 0.925, F1-score of 0.892, and AUC of 0.956 using EfficientNetV2-S. Importantly, the contribution is not limited to classification performance. The framework additionally incorporates Grad-CAM-based explainability and activation-based weak localization, allowing approximate abnormal regions to be identified without requiring bounding-box annotations during training. Fink et al. [19] further demonstrated the application of deep learning to musculoskeletal radiographs, using a patient-level dataset split and Grad-CAM heatmaps to visualize image regions influencing model predictions.

The literature therefore reveals a clear gap. Existing studies have primarily concentrated on improving classification performance, whereas explainability and localization have generally received less integrated attention. Furthermore, differences in datasets and anatomical subsets make direct performance comparisons challenging.

To address these limitations, this paper proposes an integrated deep learning framework for musculoskeletal abnormality detection using transfer learning-based models. The evaluated framework combines comparative evaluation of state-of-the-art deep learning architectures with Grad-CAM-based explainability, activation-based weak localization, and a clinically motivated sensitivity-focused decision threshold within a unified computer-aided diagnostic system. The main contributions of this work are summarized as follows:

- A comparative evaluation of four state-of-the-art deep learning architectures (DenseNet201, EfficientNetV2-S, ConvNeXt Tiny, and Swin Transformer Tiny) for musculoskeletal abnormality classification.
- Grad-CAM-based visual attribution with activation-based region visualization to generate exploratory heatmaps and approximate bounding boxes without requiring bounding-box annotations during training. Implementation of a sensitivity-focused decision threshold strategy to investigate the trade-off between sensitivity and specificity in musculoskeletal abnormality classification. An empirical decision-threshold analysis quantifying the sensitivity–specificity trade-off between a sensitivity-focused threshold of 0.40 and the conventional 0.50 baseline using identical internal validation-set predictions.

The remainder of this paper is organized as follows. Section 3 presents the proposed methodology, Section 4 discusses the experimental results, Section 5 outlines future research directions, and Section 6 concludes the paper.

## 3. Materials and Methods

**Figure 1:**
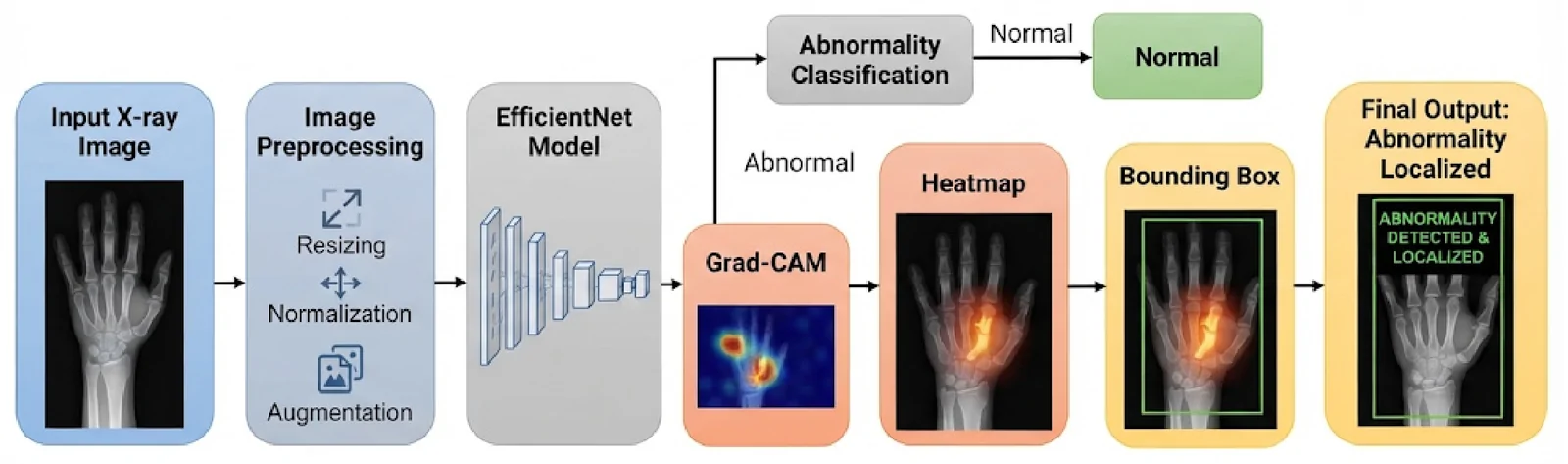
Explainable abnormality detection workflow.

Figure 1 presents the framework, which combines transfer-learning classification, Grad-CAM-based visual attribution, activation-derived bounding boxes, and a sensitivity-focused decision threshold [5,6]. The objective is to evaluate classification performance while providing exploratory visual information about regions associated with model predictions; the visualizations do not establish clinically validated pathological localization or diagnostic interpretability.

Initially, input musculoskeletal radiographs undergo image resizing, normalization, and data augmentation before being forwarded to a transfer learning-based deep learning model for binary classification (normal or abnormal). This work supports multiple transfer learning architectures, enabling comparative evaluation of DenseNet201, EfficientNetV2-S, ConvNeXt Tiny, and Swin Transformer Tiny using an identical processing pipeline [5].

To provide exploratory spatial attribution, the Grad-CAM heatmaps are further processed using an activation-based weak localization approach to generate bounding boxes around highly activated regions without requiring bounding-box annotations during training. These bounding boxes represent activation-derived regions of interest rather than validated pathological locations. Finally, a sensitivity-focused decision threshold of 0.40 is applied during inference to prioritize sensitivity, with its effect on false-negative and false-positive predictions evaluated against the conventional threshold of 0.50. The resulting framework combines abnormality classification with supplementary visual attribution and approximate activation-derived bounding boxes. However, the clinical relevance and localization accuracy of these visualizations remain unverified and require independent validation using expert-annotated radiographs and quantitative evaluation metrics [6,8,9].

### 3.1. Dataset and Data Preprocessing

This work was evaluated using the Musculoskeletal Radiographs (MURA) dataset, a publicly available benchmark dataset for musculoskeletal abnormality detection. MURA contains radiographic examinations from seven anatomical regions: the shoulder, humerus, elbow, forearm, wrist, hand, and finger [1,2]. Because MURA is organized at the study level, the provided metadata and image files were used to obtain individual radiographic images together with their corresponding anatomical-region and binary abnormality labels.

**Figure 2:**
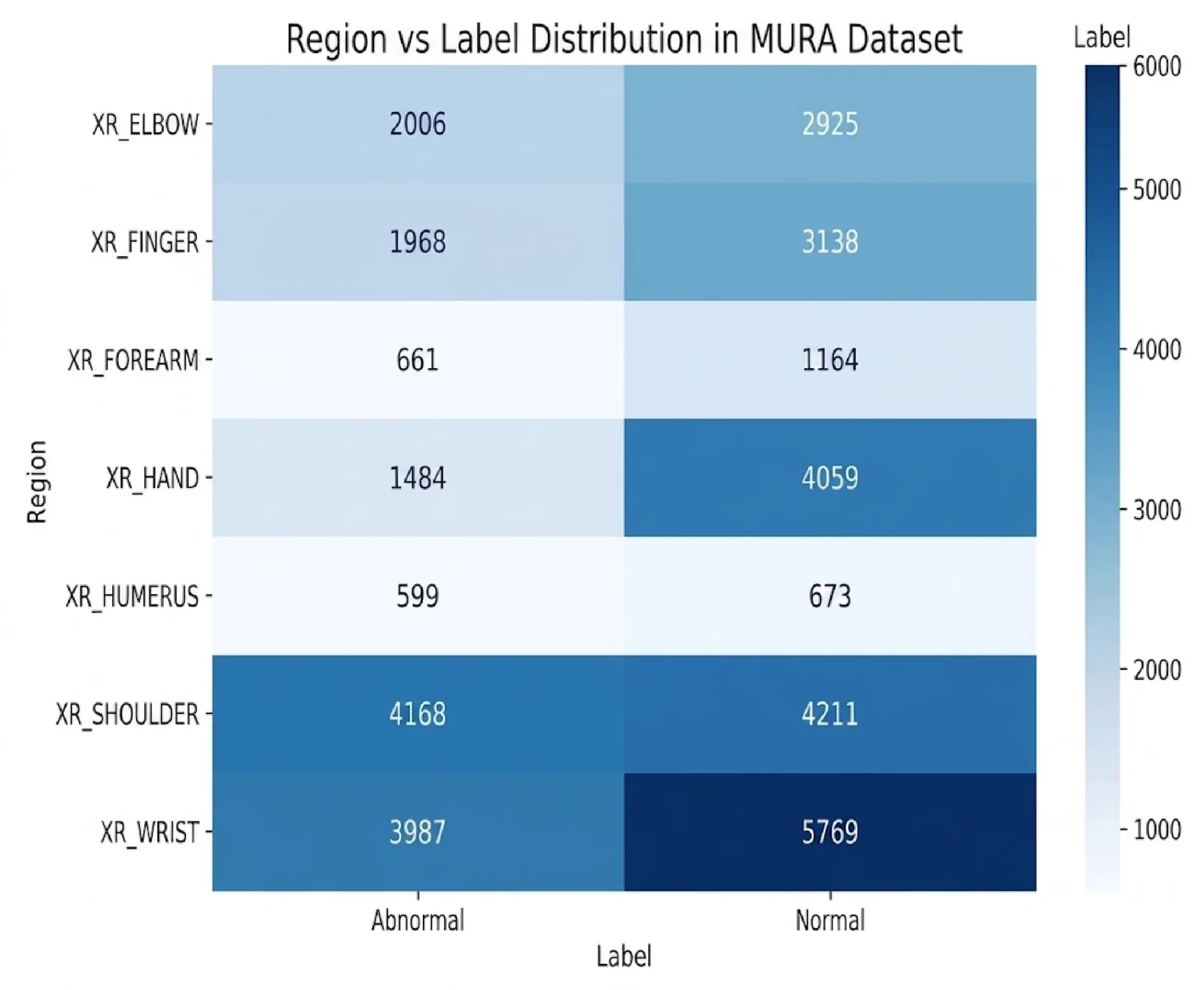
Distribution of normal and abnormal radiographs.

The image-level extraction procedure resulted in 36,812 labeled radiographs, comprising 21,939 normal and 14,873 abnormal images. Figure 2 illustrates the distribution of normal and abnormal radiographs across the seven anatomical regions in the original image-level dataset before preprocessing and balancing. The resulting class imbalance may cause a model to favor the majority class and potentially reduce its ability to identify abnormal examinations. Therefore, class balancing was incorporated into the preprocessing pipeline [20]. Detailed anatomical-region and class distributions before and after balancing are provided in Supplementary Tables S1 and S2, respectively.

Following region balancing, the intermediate dataset contained 56,000 observations, comprising 33,790 normal and 22,210 abnormal observations. Class balancing was then performed as a second and separate step. While 22,210 normal observations were chosen at random without replacement from the 33,790 normal observations that were available, all 22,210 abnormal observations were kept. As a result, 44,420 photos with equal numbers of normal and abnormal instances made up the final class-balanced dataset. Therefore, the final class-balanced dataset used for model development is 44,420, whereas the intermediate region-balanced dataset is 56,000.

A stratified 80:20 split was used to separate the final dataset of 44,420 images into training and validation subsets, producing 35,536 training images and 8884 validation images. There were 17,768 normal and 17,768 abnormal photos in the training subset and 4442 normal and 4442 abnormal images in the validation subset. The training subset was used for model optimization, whereas the validation subset was retained for model evaluation. The same data partition and preprocessing protocol were applied to all four transfer-learning architectures to ensure a consistent comparison of their classification performance.

#### 3.1.1. Data Leakage Assessment and Partition Integrity

A retrospective leakage audit was carried out on the initial training and validation partitions utilized in the presented experiments to investigate the possible risk of data leakage resulting from the anatomical-region and class-balancing methods.

To find any overlap between the two divisions, the audit looked at patient identifiers, study identifiers, original source-image paths, and image-content hashes. Furthermore, the validation subset was analyzed to see if any observations produced by oversampling were repeated in the validation data. Table S3 displays the audit results. These tests were carried out to determine whether information overlap introduced by the partitioning and resampling approach could have an impact on the claimed model performance’s dependability.

#### 3.1.2. Independent Test-Set Partitioning

To address potential model-selection bias arising from repeated use of the validation dataset, an independent held-out test set was introduced. The training and internal validation subsets were used exclusively for model development, whereas the test set was reserved for final performance evaluation.

Patient-, study-, and image-level independence between the training, validation, and test partitions was verified using the corresponding identifiers and original image paths. No oversampling, undersampling, or data augmentation was applied to the held-out test set, thereby preserving its original anatomical-region and class distributions. The test set was not used for model training, hyperparameter optimization, learning-rate scheduling, early stopping, checkpoint selection, or decision-threshold selection.

All radiographs were resized to 224 × 224 pixels and normalized using the ImageNet mean and standard deviation to match the input requirements of the pretrained transfer-learning models. During training, random horizontal flipping and small random rotations were applied as data augmentation to improve robustness and reduce overfitting [21]. These transformations were applied only to the training data while preserving the radiographic characteristics relevant to abnormality classification. No augmentation transformations were applied to the validation data. A fixed random seed of 42 was used for region resampling, class balancing, and dataset partitioning to ensure reproducibility.

### 3.2. Deep Learning Models and Training Strategy

#### 3.2.1. Model Architectures

Four state-of-the-art transfer learning architectures were evaluated to investigate the effectiveness of different deep learning paradigms for musculoskeletal abnormality classification. The selected models include DenseNet201, EfficientNetV2-S, ConvNeXt Tiny, and Swin Transformer Tiny, representing both convolutional neural network (CNN) and transformer-based architectures. These models were selected because of their strong performance on large-scale image classification benchmarks, efficient feature representation capabilities, and widespread adoption in transfer learning for medical imaging applications [3,4,22,23]. DenseNet201 employs dense feature connections that encourage feature reuse, strengthen feature propagation, and facilitate efficient gradient flow. EfficientNetV2-S improves computational efficiency through compound scaling while maintaining high classification performance. ConvNeXt Tiny modernizes conventional CNN design by incorporating transformer-inspired architectural principles, whereas Swin Transformer Tiny constructs hierarchical feature representations using shifted-window self-attention to efficiently model both local and long-range contextual information [3,4,22,23].

All models were initialized using ImageNet pretrained weights and subsequently fine-tuned on the MURA dataset under identical experimental conditions to ensure a fair and consistent comparative evaluation [5]. Supplementary Table S4 summarizes the transfer learning models evaluated in this study together with their architectural category, pretrained weights, and experimental purpose.

#### 3.2.2. Training Strategy

All models were trained using transfer learning under identical experimental settings to ensure a fair performance comparison. Cross-entropy loss [24] was employed for binary classification, while the Adam optimizer [25] was used with an initial learning rate of 1 × 10^−4^ and a batch size of 32. Each model was trained for a maximum of 10 epochs, which was sufficient to achieve model convergence under transfer learning initialization. The main training hyperparameters adopted in this study are summarized in Supplementary Table S5.

To improve optimization and reduce overfitting, a ReduceLROnPlateau learning rate scheduler [26] was employed to decrease the learning rate when the validation F1-score plateaued, together with an early stopping strategy based on the validation F1-score [27]. Mixed precision training was also adopted to improve computational efficiency and reduce GPU memory consumption [28].

Following model selection, the selected checkpoints and classification thresholds were fixed before independent test evaluation. The held-out test dataset was used exclusively to assess final classification performance. No model parameters or hyperparameters were adjusted based on the test results.

### 3.3. Gradient-Based Explainability

Grad-CAM generates weighted class activation maps from the gradients of the final convolutional layer, providing visual explanations of the model’s decision-making process [6]. Grad-CAM generates class activation maps from the gradients of the final convolutional layer, providing visual explanations of the model’s decision-making process. Such explainability is particularly important in medical imaging, where understanding the rationale behind automated predictions can improve clinical trust and transparency [7]. In this study, the generated activation maps also served as the basis for subsequent weak localization of musculoskeletal abnormalities.

The resulting heatmaps were subsequently processed to identify the regions most strongly associated with the predicted abnormality. To achieve weak localization, Grad-CAM activation maps were thresholded to retain highly activated regions, using an activation threshold of 0.6. Contour detection was then applied to identify the largest connected activation region, from which a bounding box was generated to provide an approximate localization of the abnormality. This weakly supervised approach enables spatial localization without requiring manually annotated bounding-box labels, making it computationally efficient for medical image analysis [8,9]. Since the localization is derived from class activation maps rather than pixel-level annotations, the resulting bounding boxes should be interpreted as approximate localization cues rather than precise lesion boundaries. The heatmaps and activation-derived bounding boxes provide exploratory spatial attribution: they indicate regions associated with the classification output but do not establish correspondence with pathology. Without lesion-level reference annotations, radiologist assessment, or quantitative explanation-fidelity analysis, no claim of validated localization, clinical interpretability, or diagnostic reliability is made.

### 3.4. Evaluation Metrics and Decision Threshold

Model performance was evaluated using precision, recall, the F1-score, area under the receiver operating characteristic curve (AUC), and confusion matrix analysis [10,11]. Precision measures the proportion of correctly identified abnormal cases among all positive predictions, whereas recall evaluates the ability of the model to correctly detect abnormal cases. In medical image analysis, recall is particularly important because false-negative predictions may delay diagnosis and lead to inappropriate clinical management [2,20]. The evaluation metrics used in this study are defined according to the standard formulations reported in the literature [11,29] are provided in Supplementary Section S3 (Equations (S1)–(S6)) and Table S6. The F1-score was adopted as the primary metric for model selection because it provides a balanced assessment of precision and recall, making it particularly suitable for imbalanced medical image classification problems [11,20]. In addition, the AUC was used to evaluate the discriminative capability of each model across different decision thresholds [10]. Confusion matrices were also analyzed to provide detailed insight into true positives, true negatives, false positives, and false negatives, thereby offering a comprehensive assessment of model reliability for musculoskeletal abnormality detection [11].

A threshold of 0.40 was assessed as a sensitivity-focused operating point during inference, while the traditional binary-classification threshold of 0.50 was chosen as the normal baseline. When an image’s estimated abnormality probability was higher than or equal to the chosen threshold, it was categorized as abnormal. The influence of threshold selection was isolated by applying both thresholds to the identical validation-set probabilities without retraining the model. At each threshold, the corresponding true-positive, true-negative, false-positive, and false-negative counts were computed along with accuracy, sensitivity, specificity, precision, and F1-score. Percentage points were used to report absolute performance changes. Since AUC evaluates discrimination throughout the whole range of potential thresholds and is therefore independent of a particular classification threshold, it was not computed independently for each operational point.

The Grad-CAM activation threshold of 0.60 mentioned in Section 3.3 should be separated from the categorization decision thresholds of 0.40 and 0.50. While the Grad-CAM activation threshold establishes which areas of the normalized heatmap are kept for approximate weak localization, the classification threshold establishes whether an image is classed as normal or abnormal.

## 4. Results and Discussion

### 4.1. Performance Comparison of Transfer Learning Models

The performance of the four evaluated transfer-learning models, namely DenseNet201, EfficientNetV2-S, ConvNeXt Tiny, and Swin Transformer Tiny, was compared using the validation dataset. To assess the robustness of the results with respect to stochastic training variability, each architecture was independently trained using five different random seeds (42, 123, 2024, 3407, and 7777), while maintaining the same dataset partitioning, preprocessing pipeline, and training configuration. The results are reported as mean ± standard deviation (SD) across the five independent runs. Recall, precision, F1-score, and the area under the receiver operating characteristic curve (AUC) were used for performance evaluation, with F1-score considered the primary metric because it provides a balanced assessment of precision and recall, particularly for imbalanced medical image classification. Table 2 summarizes the comparative performance of the evaluated models. The retrospective audit identified no shared patient, study, or source-image identifiers and no duplicated oversampled observations within the validation subset (Table S3).

As shown in Table 2, EfficientNetV2-S achieved the highest mean performance in terms of precision, F1-score, and AUC, with values of 0.925 ± 0.006, 0.892 ± 0.005, and 0.956 ± 0.004, respectively. These results indicate that EfficientNetV2-S provided the most balanced classification performance among the evaluated architectures under the adopted experimental configuration. Its relatively high precision, combined with a competitive recall of 0.861 ± 0.005, resulted in the highest mean F1-score. The highest mean AUC further indicates that EfficientNetV2-S provided strong discrimination between the normal and abnormal classes across different decision thresholds. This performance may be attributed to the compound scaling strategy of EfficientNetV2, which jointly balances network depth, width, and input resolution to achieve effective feature extraction while maintaining computational efficiency [22]. DenseNet201 achieved the highest mean recall of 0.876 ± 0.006, indicating that it correctly identified a comparatively larger proportion of abnormal cases. However, its lower precision of 0.873 ± 0.009 resulted in a lower mean F1-score of 0.874 ± 0.007 compared with EfficientNetV2-S. These results suggest that, although DenseNet201 demonstrated the strongest sensitivity among the evaluated models, its overall balance between precision and recall was less favorable than that of EfficientNetV2-S. ConvNeXt Tiny demonstrated competitive performance, with a mean recall of 0.862 ± 0.008, precision of 0.889 ± 0.007, F1-score of 0.876 ± 0.006, and AUC of 0.949 ± 0.005. It achieved higher mean precision, F1-score, and AUC than DenseNet201, while exhibiting a slightly lower recall. Nevertheless, its mean F1-score remained below that of EfficientNetV2-S, indicating that EfficientNetV2-S provided the strongest overall balance between precision and recall under the evaluated experimental conditions.

Swin Transformer Tiny exhibited the lowest mean performance across the principal evaluation metrics, with recall, precision, F1-score, and AUC of 0.836 ± 0.010, 0.853 ± 0.009, 0.844 ± 0.008, and 0.927 ± 0.006, respectively. The relatively larger standard deviations observed for Swin Transformer Tiny also suggest greater run-to-run variability for several metrics compared with EfficientNetV2-S. A possible explanation is that transformer-based architectures may benefit from larger training datasets and/or more extensive optimization to fully exploit their representation-learning capabilities [4]. Thus, under the dataset size and training configuration adopted in this study, Swin Transformer Tiny did not demonstrate a performance advantage over the evaluated convolutional architectures.

**Figure 3:**
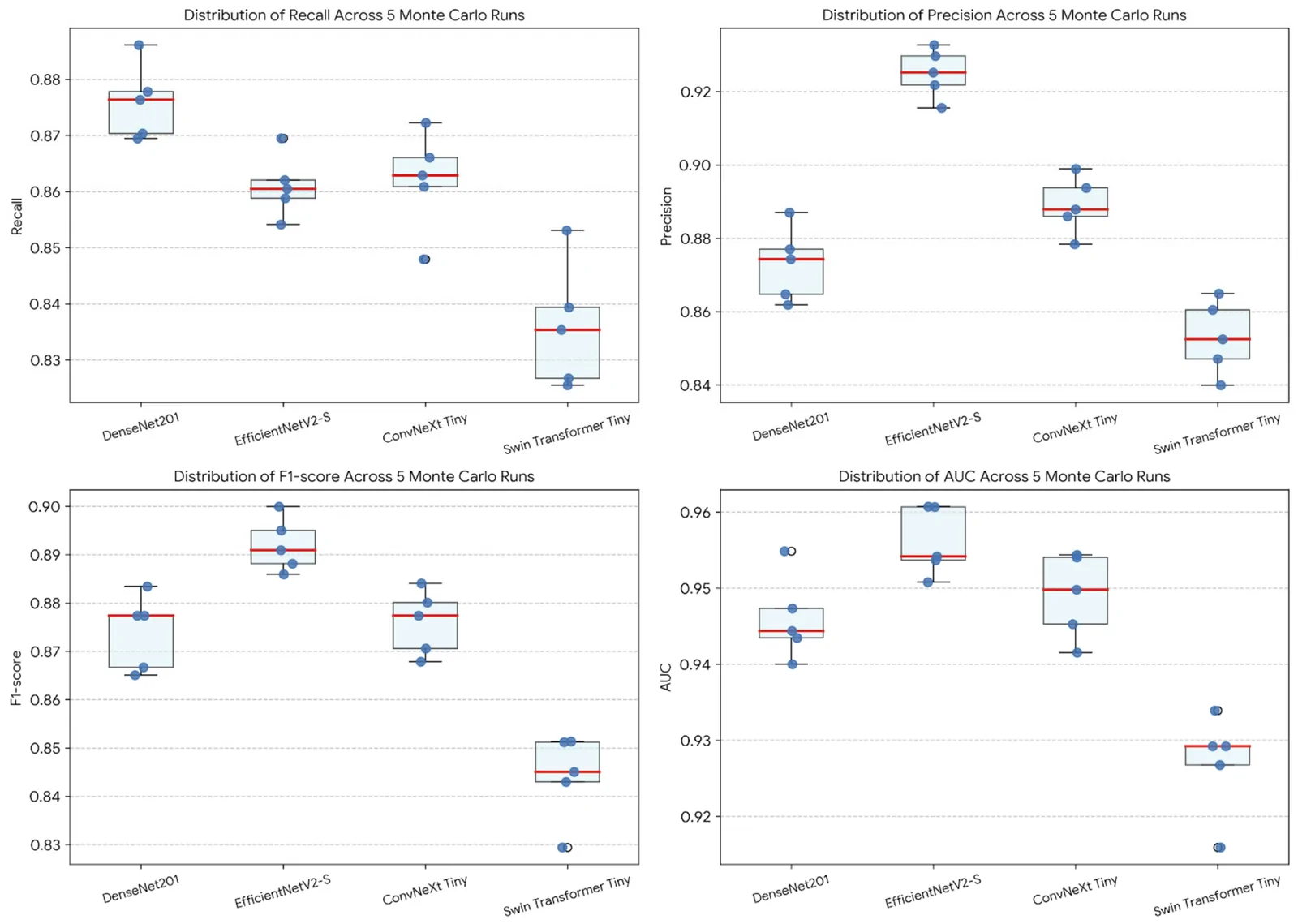
Distribution of (**Top Left**) recall, (**Top Right**) precision, (**Bottom Left**) F1-score, and (**Bottom Right**) AUC across five independent Monte Carlo runs for DenseNet201, EfficientNetV2-S, ConvNeXt Tiny, and Swin Transformer Tiny.

Figure 3 provides a visual comparison of the performance metrics reported in Table 2. The red horizontal line represents the median of each performance metric across five independent runs. The box represents the interquartile range, while the blue dots indicate individual runs. The box plots show the median and interquartile range of the performance distributions, while the individual markers represent the outcomes obtained from the five independent random seeds. The observed distributions provide an assessment of the run-to-run variability and consistency of the evaluated architectures under the adopted experimental configuration. As illustrated, EfficientNetV2-S demonstrates superior overall stability and performance across precision, F1-score, and AUC, whereas DenseNet201 achieves the highest mean recall. The resulting sensitivity, specificity, precision, F1-score, and confusion matrices were calculated independently of the validation-based threshold-selection procedure.

#### Independent Test-Set Performance

To obtain an independent assessment of classification performance, the four evaluated architectures were further assessed using the held-out test dataset, which was excluded from model development and checkpoint selection.

As presented in Table 3, EfficientNetV2-S achieved a recall of 0.826, precision of 0.886, F1-score of 0.855, and AUC of 0.927. DenseNet201 achieved a recall of 0.831, precision of 0.844, F1-score of 0.837, and AUC of 0.912. ConvNeXt Tiny obtained a recall of 0.823, precision of 0.856, F1-score of 0.839, and AUC of 0.919, while Swin Transformer Tiny achieved a recall of 0.790, precision of 0.810, F1-score of 0.800, and AUC of 0.895.

EfficientNetV2-S achieved the highest F1-score, precision, and AUC among the evaluated architectures on the independent test set, while DenseNet201 achieved the highest recall. The independent test results exhibited lower performance than the corresponding internal validation results. This difference highlights the importance of separating model selection from final performance evaluation and supports a more conservative interpretation of the framework’s generalization performance. A detailed summary of previously reported classification performance is provided in Supplementary Table S7. These results are descriptive because the studies differ in anatomical coverage, dataset composition, preprocessing, data partitions, and evaluation units. They do not establish superiority of the present approach. The controlled comparison is limited to the four architectures evaluated under the common experimental framework; comparisons with other methods would require the same predefined test set and evaluation protocol.

### 4.2. Classification Performance Analysis

**Figure 4:**
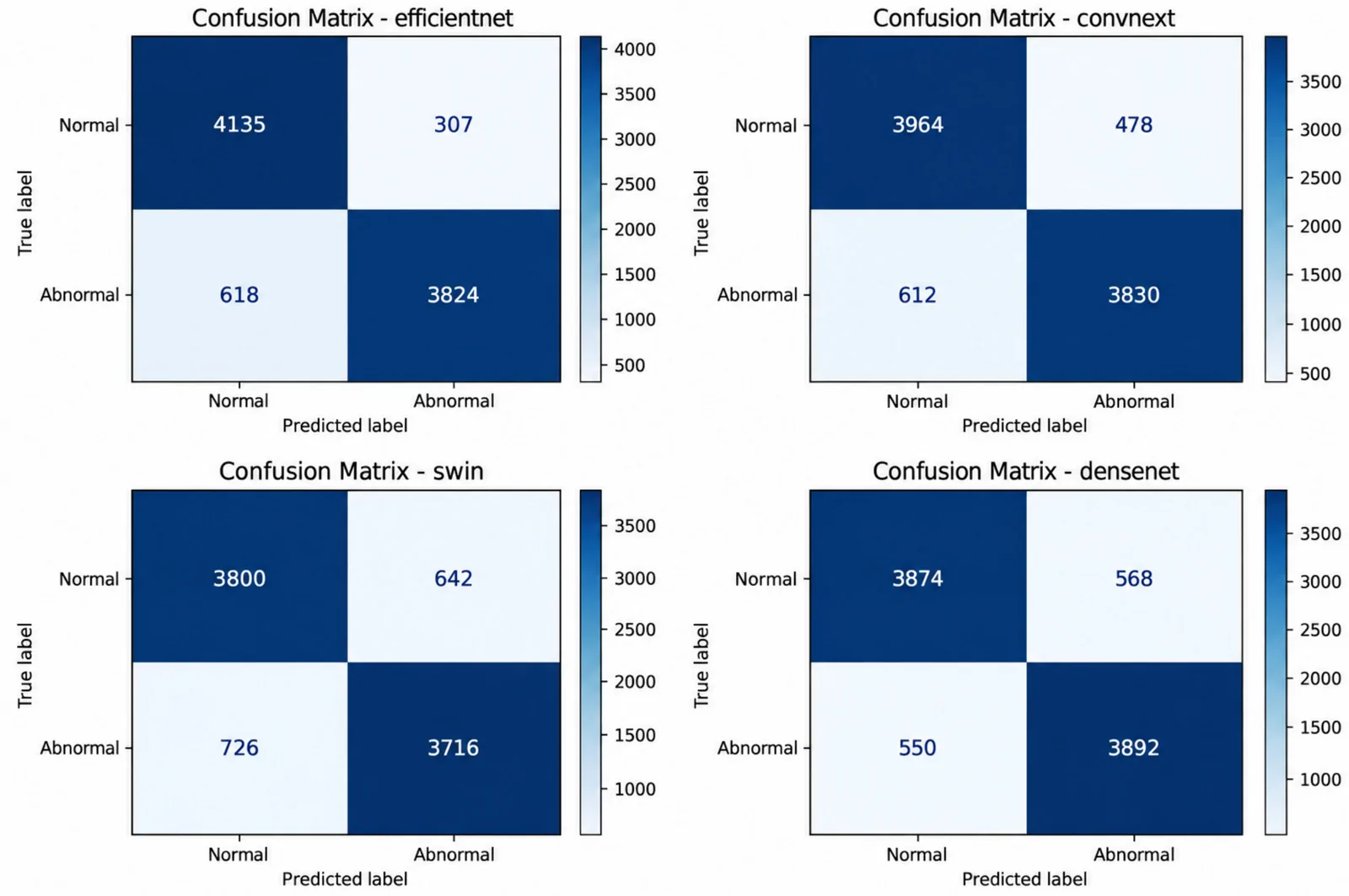
Confusion matrices of the evaluated transfer learning models.

To provide further insight into the classification behavior of the evaluated models, confusion matrices were generated for each transfer learning architecture. Figure 4 presents the confusion matrices of DenseNet201, EfficientNetV2-S, ConvNeXt Tiny, and Swin Transformer Tiny, illustrating the distribution of correctly and incorrectly classified normal and abnormal radiographs.

As shown in Figure 4, EfficientNetV2-S achieved the most balanced classification performance, correctly classifying 4135 normal and 3824 abnormal radiographs while producing the lowest number of false-positive predictions (307) among the evaluated models. This indicates a strong ability to distinguish between normal and abnormal musculoskeletal radiographs while minimizing false-positive predictions, which is consistent with its superior precision reported in Table 2.

DenseNet201 correctly identified a greater number of abnormal radiographs (3892), corresponding to fewer false-negative predictions (550), which is consistent with its highest recall reported in Table 2. However, EfficientNetV2-S substantially reduced the number of false-positive predictions (307 compared with 568 for DenseNet201), resulting in the highest precision and overall F1-score among the evaluated models.

ConvNeXt Tiny also demonstrated competitive classification performance, producing slightly fewer false-negative predictions than EfficientNetV2-S (612 vs. 618), although it generated a higher number of false-positive predictions (478 vs. 307). In contrast, Swin Transformer Tiny produced the largest number of both false-positive (642) and false-negative (726) predictions, indicating comparatively weaker classification performance under the current experimental setting.

Overall, the confusion matrix analysis is consistent with the quantitative evaluation presented in Table 2 and further confirms that EfficientNetV2-S provides the best balance between sensitivity and precision. Consequently, EfficientNetV2-S was selected for the subsequent explainability and weak localization analyses.

### 4.3. Explainability and Weak Localization Analysis

The Grad-CAM visualizations and activation-derived bounding boxes provide qualitative insight into the image regions associated with model predictions. Representative examples demonstrate the feasibility of generating spatial visualizations from image-level classification models without requiring additional lesion-level annotations during training. However, the observed activation patterns do not establish accurate localization of musculoskeletal abnormalities. In the absence of expert-annotated reference regions, localization performance could not be quantified using metrics such as Intersection over Union (IoU), Dice similarity, or localization accuracy. The generated bounding boxes should therefore be interpreted as approximate activation-derived regions of interest rather than validated pathological locations.

**Figure 5:**
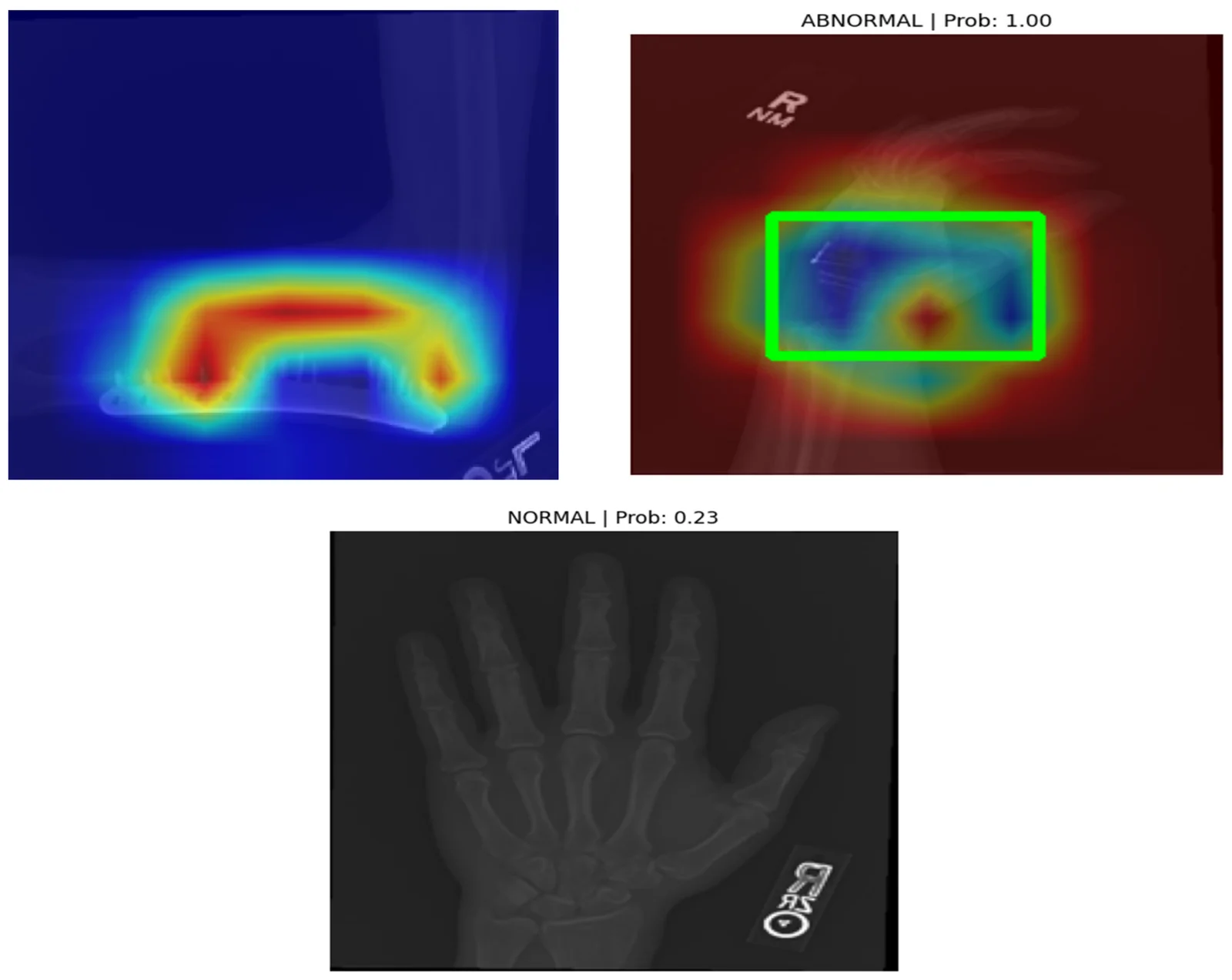
Comparison of Grad-CAM responses for representative normal and abnormal radiographs.

The Grad-CAM analysis provides qualitative visual information regarding regions associated with model predictions. Representative examples illustrate the generation of activation maps and corresponding bounding boxes for musculoskeletal radiographs (Figure 5).

However, anatomical plausibility alone does not establish that the model identifies pathological findings or relies on clinically meaningful features. In particular, activation may arise from normal anatomical structures, image artifacts, or other features correlated with the classification label. Since the present study did not include lesion-level reference annotations, blinded radiologist assessment, or quantitative explanation-fidelity analysis, the clinical relevance and reliability of the generated heatmaps remain unverified. Consequently, the Grad-CAM results should be regarded as exploratory visual attribution rather than validated evidence of pathological localization, diagnostic interpretability, or clinical trustworthiness.

### 4.4. Decision-Threshold Ablation Analysis

A decision-threshold ablation analysis was carried out to measure the impact of threshold selection on the top-performing EfficientNetV2-S model. The traditional binary classification threshold of 0.50 was contrasted with the sensitivity-focused criterion of 0.40. The impact of altering the operational threshold was isolated by applying both thresholds to the identical validation-set probability without retraining the model. The evaluation of 0.40 was motivated by the importance of reducing false-negative predictions in abnormality screening, where missing an abnormal examination may have more serious consequences than generating an additional false-positive prediction [10,11].

Table 4 illustrates that EfficientNetV2-S obtained an accuracy of 0.860, sensitivity of 0.754, specificity of 0.966, precision of 0.957, and F1-score of 0.844 at the standard threshold of 0.50. There were 3350 true-positive, 4292 true-negative, 150 false-positive, and 1092 false-negative predictions in the related confusion matrix with.

The model obtained an accuracy of 0.896, sensitivity of 0.861, specificity of 0.931, precision of 0.925, and F1-score of 0.892 at the sensitivity-focused threshold of 0.40. There were 3824 true-positive, 4135 true-negative, 307 false-positive, and 618 false-negative predictions in the matching confusion matrix. Sensitivity increased by 10.7 percentage points, from 0.754 to 0.861, when the decision threshold was lowered from 0.50 to 0.40. This represents a relative improvement of 14.2%. From 1092 to 618, the number of false-negative predictions dropped by 474, or 43.4%. The F1-score rose by 4.8 percentage points, from 0.844 to 0.892, while accuracy climbed by 3.6 percentage points, from 0.860 to 0.896.

These improvements were accompanied by the expected sensitivity–specificity trade-off. Precision dropped by 3.1 percentage points, from 0.957 to 0.925, while specificity dropped by 3.5 percentage points, from 0.966 to 0.931. From 150 to 307, there were 157 more false-positive predictions. Consequently, at the expense of more false-positive predictions, the threshold of 0.40 enhanced the identification of abnormal photos and significantly decreased missed abnormal cases.

The AUC remained 0.956 at both operating points because it is calculated across the complete range of possible thresholds and is therefore independent of any single classification threshold. Consequently, AUC is reported once for EfficientNetV2-S rather than separately for each row in Table 4.

The decision-threshold ablation concerns only the conversion of predicted abnormality probabilities into binary classifications. It should be distinguished from the weak-localization procedure described in Section 4.3, which uses a separate activation threshold to identify highly activated regions within Grad-CAM heatmaps. The Grad-CAM-derived regions provide approximate spatial explanations and do not affect the predicted probabilities or classification performance [6,7,8,9].

Overall, the results support interpreting 0.40 as a sensitivity-oriented operating point rather than a universally optimal decision threshold. Its suitability depends on the relative clinical consequences of missed abnormalities and unnecessary referrals. Further evaluation using independent clinical datasets and clinically defined error costs is required before recommending a specific operating threshold for clinical application.

### 4.5. Error Analysis

Despite the strong overall performance achieved by EfficientNetV2-S, a number of misclassified cases were observed during model evaluation. Examination of the confusion matrices presented in Figure 4 indicates that most prediction errors occurred in challenging radiographs containing subtle abnormalities, overlapping anatomical structures, poor image contrast, or equivocal fracture patterns. Such cases remain inherently difficult for both deep learning models and human observers [2].

False-negative predictions were primarily associated with subtle pathological findings that generated relatively weak activation patterns in the Grad-CAM visualizations. In contrast, false-positive predictions were more commonly related to normal anatomical variations, image artifacts, or complex structural appearances that produced activation in regions resembling abnormal findings. These observations are also consistent with the qualitative analyses presented in Section 3.3 and further highlight the challenges associated with musculoskeletal abnormality detection [6,7].

Overall, the error analysis complements the quantitative evaluation by providing insight into the practical limitations of the studied framework that cannot be fully captured using conventional performance metrics. Nevertheless, the evaluated EfficientNetV2-S model demonstrated robust and well-balanced performance across the MURA (all seven anatomical regions), confirming its effectiveness for musculoskeletal abnormality detection.

### 4.6. Study Limitations

Although the introduction of an independent held-out test set reduces the potential model-selection bias associated with validation-based performance reporting, the present study remains limited to evaluation within the MURA dataset. The observed differences between internal validation and independent test performance suggest that estimates obtained during model development may not fully reflect generalization performance. Furthermore, independent testing within the same dataset does not establish generalizability across institutions, imaging protocols, or patient populations. Future studies will investigate external validation using multicenter datasets and prospective clinical evaluation to further assess the robustness and clinical applicability of the proposed framework.

The activation-based region visualization component was evaluated qualitatively using representative radiographs. The absence of lesion-level ground-truth annotations prevented quantitative evaluation of localization performance using metrics such as IoU, Dice similarity, and localization accuracy. Furthermore, the generated regions were not independently assessed by radiologists to establish their correspondence with clinically relevant abnormalities. Therefore, the spatial visualizations should not be interpreted as validated lesion-localization predictions. Future investigations will incorporate expert-annotated radiographs and quantitative localization metrics to assess the accuracy and clinical relevance of the activation-derived regions. An additional limitation concerns the evaluation of the explainability component. Although Grad-CAM provided visual attribution maps and activation-derived regions, the present study did not include blinded radiologist assessment, lesion-level ground-truth annotations, or quantitative explanation-fidelity metrics.

Consequently, the anatomical plausibility of the highlighted regions cannot be interpreted as evidence that the model consistently attends to pathological findings. Similarly, the generated heatmaps and bounding boxes cannot establish clinical interpretability, localization accuracy, or diagnostic trustworthiness.

Future work will incorporate expert-annotated radiographs, blinded radiologist assessments, systematic analysis of explanation failures, and quantitative explanation metrics to evaluate the clinical relevance and reliability of the proposed visualizations.

## 5. Future Work

Although the evaluated framework demonstrated robust performance for musculoskeletal abnormality detection, several directions remain for future investigation.

First, future studies should evaluate the evaluated framework using larger, more diverse, and multicenter datasets to further assess its robustness and generalizability across different imaging protocols, patient populations, and clinical environments. In addition, external validation on independent datasets would provide a more comprehensive assessment of model reliability prior to clinical deployment, as highlighted in recent musculoskeletal imaging studies [10,15].

Second, although the Grad-CAM-based weak localization approach provides useful visual explanations without requiring additional annotations, more advanced explainability and localization techniques could be investigated to improve both interpretability and localization accuracy. For example, integrating Grad-CAM++, occlusion sensitivity, or supervised detection and segmentation methods may provide more precise delineation of abnormal regions while maintaining clinically meaningful visual explanations [6,7,8,9,15].

Although the present analysis quantified the performance difference between the sensitivity-focused threshold of 0.40 and the conventional 0.50 baseline, future studies should evaluate a denser range of operating thresholds and report uncertainty estimates using independent test data. Threshold selection should also incorporate clinically defined costs for false-negative and false-positive predictions and be externally validated across different patient populations and imaging environments.

Finally, prospective clinical validation involving radiologists is necessary to further assess the practical usefulness and clinical acceptance of the studied framework within real-world diagnostic workflows. Rather than replacing expert clinical judgment, the evaluated framework is intended to function as a computer-aided diagnostic support tool that assists clinicians by combining accurate abnormality detection with interpretable visual explanations [15,30].

## 6. Conclusions

This study presented a deep learning framework incorporating Grad-CAM-based visual attribution for automated musculoskeletal abnormality detection using the MURA dataset across all seven anatomical regions. Four transfer-learning architectures—DenseNet201, EfficientNetV2-S, ConvNeXt Tiny, and Swin Transformer Tiny—were systematically evaluated using a common experimental protocol. Among the evaluated models, EfficientNetV2-S achieved the highest F1-score (0.892), precision (0.925), and AUC (0.956), while DenseNet201 achieved the highest recall (0.876) on the internal validation set. EfficientNetV2-S also achieved an F1-score of 0.855 and an AUC of 0.927 on the independent held-out test set, providing a separate assessment of its classification performance.

Grad-CAM heatmaps and activation-derived bounding boxes provided exploratory spatial attribution without lesion-level supervision. Their localization accuracy and clinical relevance remain unverified because expert annotations, blinded radiologist assessment, and explanation-fidelity analyses were unavailable. On the internal validation set, lowering the classification threshold from 0.50 to 0.40 increased sensitivity from 0.754 to 0.861 and reduced false negatives from 1092 to 618, while specificity decreased from 0.966 to 0.931 and false positives increased from 150 to 307. This operating point therefore represents a sensitivity–specificity trade-off rather than a universally optimal threshold. Cross-study performance differences do not establish superiority because evaluation protocols vary. External validation, controlled comparisons on common test sets, quantitative evaluation of localization and explanations, and clinically informed threshold selection are required before clinical use [ER10.1].

## Figures and Tables

**Table 1:** Summary of representative studies on musculoskeletal abnormality detection.

Ref.	Dataset	Model/Method	Recall	Precision	F1-Score	AUC	Main Task
[1]	MURA	DenseNet-169	0.815	—	—	0.929	Abnormality classification
[15]	MURA	Two-stage VGG16	—	—	0.688	N/R	Abnormality classification
[16]	MURA + LERA	Hybrid CNN + AE + SE	0.860	—	—	0.940	Hybrid classification
[17]	MURA	Two-stage DenseNet201	0.690	0.870	0.790	0.790	Abnormality classification
[18]	MURA (Shoulder)	Transfer learning + feature fusion	0.996	0.989	0.992	—	Shoulder abnormality classification
This study	MURA	EfficientNetV2-S + Grad-CAM + Weak Localization	0.861	0.925	0.892	0.956	Classification + Explainability + Localization

**Table 2:** Internal validation performance of the evaluated transfer-learning architectures.

Model	Recall (Mean ± SD)	Precision (Mean ± SD)	F1-Score (Mean ± SD)	AUC (Mean ± SD)
DenseNet201	0.876 ± 0.006	0.873 ± 0.009	0.874 ± 0.007	0.946 ± 0.005
EfficientNetV2-S	0.861 ± 0.005	0.925 ± 0.006	0.892 ± 0.005	0.956 ± 0.004
ConvNeXt Tiny	0.862 ± 0.008	0.889 ± 0.007	0.876 ± 0.006	0.949 ± 0.005
Swin Transformer Tiny	0.836 ± 0.010	0.853 ± 0.009	0.844 ± 0.008	0.927 ± 0.006

**Table 3:** Independent test performance of the evaluated transfer-learning architectures.

Model	Recall	Precision	F1-Score	AUC
DenseNet201	0.831	0.844	0.837	0.912
EfficientNetV2-S	0.826	0.886	0.855	0.927
ConvNeXt Tiny	0.823	0.856	0.839	0.919
Swin Transformer Tiny	0.790	0.810	0.800	0.895

**Table 4:** Decision-threshold ablation analysis for EfficientNetV2-S.

Model/Threshold	Accuracy	Sensitivity	Specificity	Precision	F1-Score	True Positive	True Negative	False Positive	False Negative
Threshold 0.40	0.896	0.861	0.931	0.925	0.892	3824	4135	307	618
Threshold 0.50 (baseline)	0.860	0.754	0.966	0.957	0.844	3350	4292	150	1092

## Data Availability

The dataset analyzed in this study is the publicly available Musculoskeletal Radiographs (MURA) dataset developed by the Stanford Machine Learning Group and distributed through the Stanford Center for Artificial Intelligence in Medicine and Imaging (AIMI). This dataset is accessible at https://aimi.stanford.edu/datasets/mura-msk-xrays (accessed on 5 march 2026). Dataset DOI: https://doi.org/10.71718/cwh3-0p32 (accessed on 5 March 2026). The original dataset and its collection methodology are described by Rajpurkar et al. [1]. This study constitutes a secondary analysis of existing de-identified radiographic data. No new patient data were collected. The dataset is available for non-commercial research subject to the applicable terms and conditions established by the data provider. Users should consult the official repository for the current access requirements and permitted uses.

## Supplementary Materials

The following supporting information can be downloaded at: https://www.mdpi.com/article/10.3390/bioengineering13101177/s1, Tables S1–S7 provide supporting dataset, model, training, evaluation-metric, and literature-comparison details. Equations (S1)–(S6) provide the evaluation-metric definitions.

## Abbreviations

The following abbreviations are used in this manuscript:

Abbreviation	Definition
AE	Autoencoder
AI	Artificial Intelligence
AUC	Area Under the Receiver Operating Characteristic Curve
CAD	Computer-Aided Diagnosis
CNN	Convolutional Neural Network
F1-score	F1-Score
Grad-CAM	Gradient-weighted Class Activation Mapping
LERA	Longitudinal Emergency Radiology Dataset
MURA	Musculoskeletal Radiographs
SE	Squeeze-and-Excitation
XAI	Explainable Artificial Intelligence
