# Supplementary material for: An Explainable Deep Learning Framework for Musculoskeletal Abnormality Detection
Source: Bioengineering (Basel). 2026 Oct 9;13(10):1177. doi: 10.3390/bioengineering13101177 (PMC13701356; doi:10.3390/bioengineering13101177)
Supplement: Supplementary file 1 [file bioengineering-13-01177-s001.zip › bioengineering-4554058-supplementary.pdf]

Supplementary material

An Explainable Deep Learning Framework for Musculoskeletal Abnormality Detection

This supplement provides supporting dataset, model, training, and evaluation details, together with the detailed literature comparison. Reference numbers follow the main manuscript.

S1 Dataset composition and balancing

Anatomical-region balancing produced 56,000 observations. Subsequent class balancing produced 44,420 observations, partitioned into 35,536 training and 8,884 internal validation images. The tables below describe these model-development data.

Table S1. Distribution of images before and after anatomical-region balancing.

Anatomical Region	Original Images	Region-Balanced Images	Operation
Elbow	4,931	8,000	Oversampling with replacement
Finger	5,106	8,000	Oversampling with replacement
Forearm	1,825	8,000	Oversampling with replacement
Hand	5,543	8,000	Oversampling with replacement
Humerus	1,272	8,000	Oversampling with replacement
Shoulder	8,379	8,000	Undersampling without replacement
Wrist	9,756	8,000	Undersampling without replacement
Total	36,812	56,000	—

Table S2. Class distribution before and after dataset balancing.

Stage	Normal	Abnormal	Total
After region balancing	33,790	22,210	56,000
After class balancing	22,210	22,210	44,420
Training subset	17,768	17,768	35,536
Validation subset	4,442	4,442	8,884

S2 Model architectures and Methods

Table S3 displays the audit results.

Table S3. Data leakage audit results

	Audit result
Shared patient identities	0
Shared study identities	0
Shared original source images	0
Shared image-content hashes	0
Oversampled validation/test observations	0

All four models used ImageNet-pretrained weights and the common training configuration summarised below. The internal validation F1-score guided scheduling, early stopping, and checkpoint selection.

Table S4. Characteristics of the evaluated transfer-learning models.

Model	Architecture Type	Pretrained Weights	Experimental Role
DenseNet201	CNN	ImageNet	Comparative evaluation
EfficientNetV2-S	CNN	ImageNet	Comparative evaluation
ConvNeXt Tiny	CNN	ImageNet	Comparative evaluation
Swin Transformer Tiny	Vision Transformer	ImageNet	Comparative evaluation

Table S5. Summary of training hyperparameters.

Parameter	Value
Input size	224 × 224
Batch size	32
Epochs	10
Optimizer	Adam
Learning rate	1×10^{-4}
Loss function	Cross-Entropy
Scheduler	ReduceLROnPlateau
ReduceLROnPlateau patience	2 epochs
Early stopping patience	3 epochs
Scheduler factor	0.5
Mixed precision training	Enabled

S3 Evaluation metric definitions

The abnormal class is the positive class. TP, TN, FP, and FN denote true positives, true negatives, false positives, and false negatives, respectively. The standard definitions below follow [11,21]; recall and sensitivity are equivalent.

$$\text{Precision} = \frac{\text{TP}}{\text{TP+FP}} \quad \text{(S1)}$$

$$\text{Recall} = \frac{\text{TP}}{\text{TP+FN}} \quad \text{(S2)}$$

$$\text{F1} - \text{score} = \frac{2\times\text{Precision}\times\text{Recall}}{\text{Precision+Recall}} \quad \text{(S3)}$$

$$\text{Sensitivity} = \frac{TP}{TP+FN} \quad \text{(S4)}$$

$$\text{Accuracy} = \frac{TP+TN}{TP+FN+TN+FP} \quad \text{(S5)}$$

$$\text{Specificity} = \frac{TN}{TN+FP} \quad \text{(S6)}$$

Table S6. Evaluation metrics and their purposes.

Metric	Purpose
Precision	Accuracy of positive predictions.
Recall	Ability to correctly identify abnormal cases.
F1-score	Balances precision and recall.

Metric	Purpose
AUC	Discrimination performance across decision thresholds.
Confusion Matrix	Summarizes TP, TN, FP, and FN predictions.
Accuracy	Proportion of all predictions classified correctly.
Sensitivity/Recall	Proportion of abnormal images correctly identified.
Specificity	Proportion of normal images correctly identified.

AUC measures discrimination across decision thresholds; it is not recalculated separately for each fixed operating point. The Grad-CAM activation threshold is distinct from the classification decision threshold.

S4 Classification results from previous studies

The results in Table S6 provide context and are not a controlled comparison. Anatomical coverage, datasets, preprocessing, data partitions, and evaluation units differ across studies. The present-work row reports internal validation results; the independent test results are reported separately in Table 4 of the main manuscript.

Table S7. Classification performance in previous studies and the present work.

Ref.	Dataset	Model	Recall	Precision	F1-score	AUC	Remarks
[1]	MURA	DenseNet-169	0.815	–	–	0.929	Original baseline
[22]	MURA	VGG16	–	–	0.688	N/R	Two-stage framework
[23]	MURA + LERA	Hybrid CNN with ResNet50 + DenseNet121 + VGG16 + Autoencoder + SE blocks	0.86	–	–	0.94	Combined MURA + LERA dataset; Accuracy = 89%, Specificity = 0.89.
[24]	MURA	Two-stage DenseNet201	0.69	0.87	0.79	0.79	Shoulder classification
[25]	Shoulder subset of MURA	Same-domain TL + Feature Fusion	0.996	0.989	0.992	–	Accuracy = 99.2%; shoulder only, not full MURA.
This work	MURA	EfficientNetV2-S	0.861	0.925	0.892	0.956	Internal validation; all seven anatomical regions of MURA

N/R denotes not reported; dashes indicate values not provided in the source comparison. TL, transfer learning; SE, squeeze-and-excitation.
